# Post-Mortem Poisoning

**Published:** 1871-09

**Authors:** 


### Post-mortem Poisoning.

It is believed that Dr. O. C. Gibbs, of Frewsburg, whose brief obituary notice appeared in our last Journal, came to his death from *post mortem* poisoning He survived the injury since April last, and hopes of his recovery were, at times, entertained; but alas! he fell another sacrifice, self-offered in the pursuit of medical knowledge.

